# Consistent pre-stimulus influences on auditory perception across the lifespan

**DOI:** 10.1016/j.neuroimage.2018.10.085

**Published:** 2019-02-01

**Authors:** Steven W. McNair, Stephanie J. Kayser, Christoph Kayser

**Affiliations:** a Institute of Neuroscience and Psychology, University of Glasgow, 62 Hillhead Street, G12 8QB, United Kingdom; b Department for Cognitive Neuroscience, Faculty of Biology, Bielefeld University, Universitätsstr. 25, 33615, Bielefeld, Germany; c Cognitive Interaction Technology – Center of Excellence, Bielefeld University, Inspiration 1, 33615, Bielefeld, Germany

**Keywords:** Aging, EEG, Single-trial decoding, Oscillatory brain activity, Pre-stimulus, Auditory evoked potential

## Abstract

As we get older, perception in cluttered environments becomes increasingly difficult as a result of changes in peripheral and central neural processes. Given the aging society, it is important to understand the neural mechanisms constraining perception in the elderly. In young participants, the state of rhythmic brain activity prior to a stimulus has been shown to modulate the neural encoding and perceptual impact of this stimulus – yet it remains unclear whether, and if so, how, the perceptual relevance of pre-stimulus activity changes with age. Using the auditory system as a model, we recorded EEG activity during a frequency discrimination task from younger and older human listeners. By combining single-trial EEG decoding with linear modelling we demonstrate consistent statistical relations between pre-stimulus power and the encoding of sensory evidence in short-latency EEG components, and more variable relations between pre-stimulus phase and subjects’ decisions in longer-latency components. At the same time, we observed a significant slowing of auditory evoked responses and a flattening of the overall EEG frequency spectrum in the older listeners. Our results point to mechanistically consistent relations between rhythmic brain activity and sensory encoding that emerge despite changes in neural response latencies and the relative amplitude of rhythmic brain activity with age.

## Highlights

• Younger and older adults completed a frequency in noise discrimination task.
• Influences of pre-stimulus activity were consistent across groups.
• Despite changes in the slope of EEG spectral density between groups.
• And despite changes in evoked response latencies and amplitudes between groups.

## 1 Introduction

In everyday life our acoustic environments are often teeming with incoming information. Yet, the auditory brain manages to filter target information from noise seamlessly, at least in the young and healthy brain (Bregman, 1994). With advancing age listening becomes more challenging, particularly in “cocktail party” scenarios (de Villers-Sidani et al., 2010; Pichora-Fuller et al., 2017; Rossi-Katz and Arehart, 2009). This difficulty could arise from age-related changes in peripheral and central auditory processes (Anderson et al., 2013; Clinard et al., 2010; Clinard and Cotter, 2015; Harris and Dubno, 2017), such as the poorer encoding in early sensory regions (Grose and Mamo, 2012; He et al., 2007; Mahajan et al., 2017; Paraouty et al., 2016; Wallaert et al., 2016). Changes in higher cognitive processes may also influence older adults’ performance via top-down feedback (Henry et al., 2017), through reduced attentional flexibility (Nunez et al., 2015; Zanto and Gazzaley, 2014), or changes in decision criteria when reporting perceptual performance (Dully et al., 2018).

As shown by recent work, perception depends not only on the qualities of the sensory signal but also on the state of the brain prior to stimulus occurrence (Henry et al., 2017, 2014; Henry and Obleser, 2012; Kayser et al., 2016; Ng et al., 2012; Pinheiro et al., 2017; Steinmetzger and Rosen, 2017). In many studies, the state (power or phase) of pre-stimulus rhythmic brain activity has been predictive of perceptual performance in a variety of tasks, in line with the view that perception in general is controlled by a cascade of rhythmic neural processes (Schroeder et al., 2010; VanRullen, 2016). Furthermore, changes in top-down influences by attentional and cognitive strategies are also reflected in rhythmic brain activity, especially in the alpha and beta bands (Henry et al., 2017; Petersen et al., 2015; Strauβ et al., 2015; Wöstmann et al., 2017). In this context of relating rhythmic brain activity to perception we recently described two putative mechanisms by which pre-stimulus activity shapes auditory perceptual decisions in younger adults (Kayser et al., 2016): in that study the power of low-frequency and beta activity affected the encoding of sensory information in early auditory regions, while the phase of the alpha band influenced decision processes in high-level regions.

This importance of rhythmic activity for perception raises the question as to whether the underlying mechanisms and relevant time scales are conserved across the age span. For example, it is known that cognitive and neural processes become slower with age (Bieniek et al., 2013; Price et al., 2017; Salthouse, 1996), which is reflected in changes in the amplitude and latency of auditory evoked responses (Harris et al., 2008; Henry et al., 2017; Tremblay et al., 2003), an increase in response stereotypy (Garrett et al., 2013, 2011; Herrmann et al., 2016), and changes in the slope of the frequency spectrum of brain activity (Hong and Rebec, 2012; Tran et al., 2016; Voytek et al., 2015). This makes it possible that the patterns of rhythmic brain activity that shape perception systematically change with age.

We here capitalized on our previous study in a group of younger subjects to directly probe whether the mechanisms linking pre-stimulus brain activity, sensory encoding and decision-making are conserved with age. Specifically, we compared behavioural and electroencephalogram (EEG) data from younger (<30 years) and older (>65 years) listeners with no, or only mild hearing loss, obtained during an auditory frequency discrimination-in-noise task. For each group we linked pre-stimulus oscillatory activity to neural signatures of stimulus encoding and decision making using single trial modelling. We expected to observe the same patterns of statistical relations between neural activity, sensory encoding and behavioural responses in both groups (i.e. significant relations between the same variables), but with the possibility that the precise time scales (i.e. frequency bands of brain activity) differed. For comparison with previous studies, we also quantified age-related changes in the amplitude and timing of evoked responses and the spectral slope of the overall EEG signal.

## 2 Materials and methods

### 2.1 Participants

We collected data from 16 younger (6 male; mean ± SD age, 23.9 ± 1.1 years) and 17 older adults (8 male; mean ± SD age, 68.4 ± 3.6 years). We have reported data from the younger group, with the exclusion of power spectral density (PSD) and auditory evoked potential (AEP) analyses, in our previous study Kayser et al. (2016) (the frequency task there). For this reason, we had set the target sample size for the group of older subjects to match the size of the younger group. Younger participants had normal self-reported hearing, as measured by the Better Hearing Institute Quick Hearing Questionnaire (Kochkin and Bentler, 2010). Older participants had no more than mild hearing loss as measured by the Better Hearing Institute Quick Hearing Questionnaire, Tinnitus Handicap Inventory (THI, where applicable; McCombe et al., 2001) and pure-tone audiometry (PTA). The PTA procedure was presented via MATLAB (2015b; The MathWorks Inc., Natick, MA) and was designed in accordance with guidelines from the British Society of Audiology (BSA, British Society of Audiology, 2012). We tested participants' hearing thresholds at frequencies of 250 Hz, 500 Hz, 1000 Hz, 2000 Hz, 4000 Hz and 8000 Hz individually for each ear. Sound levels were calibrated using a Bruel&Kjaer sound-level meter. Older participants were also screened for cognitive impairment using the Montreal Cognitive Assessment (MoCA, Nasreddine et al., 2005), D2 test of attention (Brickenkamp and Zillmer, 1998), and the digit span working memory test (Turner and Ridsdale, 2004). Due to possible variability in participants’ frequency discrimination abilities (Foxton et al., 2009; Liang et al., 2016; Semal and Demany, 2006), frequency difference limens (see below) were tested both at screening and immediately prior to the main experiment for each group. Group-level auditory and cognitive test scores are shown in Table 1. Four older participants were excluded at screening based on pre-defined criteria: two participants had moderate to severe hearing loss, as indicated by PTA testing, and in two participants frequency difference limens could not be measured reliably. Participants indicated no history of mental/neuropsychological disorders, stroke, or brain or ear injuries. Participants gave written informed consent and received £6/hour payment plus travel expenses for participating. This study is in accordance with the Declaration of Helsinki and was approved by the local ethics committee (College of Science and Engineering, University of Glasgow).

**Table 1:** Auditory and cognitive test scores. Screening scores for younger (where applicable) and older participants who passed screening. Hearing scores are derived from pure tone audiometry (PTA), Better Hearing Institute Quick Hearing Questionnaire (BHI QHQ), and Tinnitus Handicap Inventory (THI). PTA scores reported are measured in decibels (dB) and represent the average threshold across ears and frequencies. THI was administered only as applicable, thus n is reported. Cognitive test scores are derived from Montreal Cognitive Assessment (MoCA), D2 test of Attention (D2) and digit span (DSpan) tests. Scores correspond to median across all participants in each age group. Square brackets indicate minimum and maximum scores. N/A indicates where data was not available.

	PTA (dB)	BHI QHQ	THI	MoCA	D2			DSpan
					CP	TN-E		
Older	28.96 [18.93, 39.72]	7 [0, 33]	6 [2, 12] (n = 3)	29 [26, 30]	199 [163, 251]	121 [113, 130]	105.5 [79, 121]	
Younger	N/A	2.5 [0, 11]	N/A	N/A	N/A	N/A	N/A	

### 2.2 Auditory stimuli

**Fig. 1:**
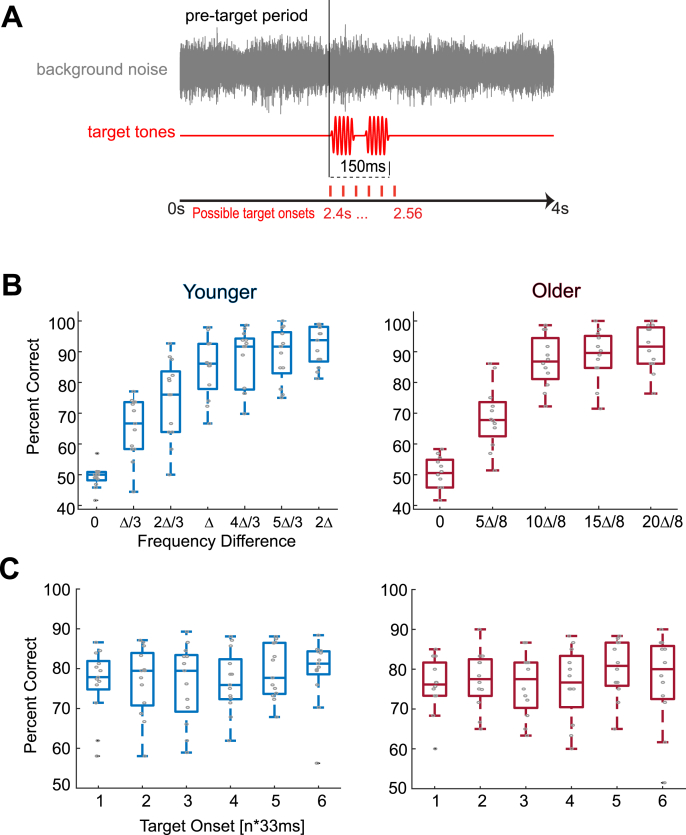
Auditory paradigm and task performance. (A) Auditory paradigm. Pure tone targets (50 ms duration, spaced 50 ms apart), were presented at one of six possible onsets against a continuous background noise cacophony. The second tone was kept at 1024 Hz while the first varied over 7 (younger adults) or 5 (older adults) levels of frequency difference, titrated around participants' own frequency difference limens, Δ. (B) Group level task performance as a function of stimulus level, averaged across target positions. Younger and older adults show comparable task performance. (C) Group level task performance as a function of target position, averaged across stimulus levels. There were no significant effects of target position on performance in either group and overall there was no significant difference between groups (across stimulus levels and target positions). Grey circles indicate individual subject data.

Participants completed a 2-alternative forced-choice auditory frequency discrimination task, as described in Kayser et al. (2016). Participants were presented with two sequential target tones embedded within a noisy background and had to discriminate which tone was higher in frequency (see Fig. 1A). Targets were pure-tones of 50 ms duration (including a 5 ms cosine on/off ramp) and spaced 50 ms apart. The noise was 4s in duration and comprised a cacophony of naturalistic sounds, consisting of environmental (forest and city) sounds, animal sounds, and sounds originating from tools (also used in Kayser et al., 2016; Ng et al., 2012). The same noise clip was used in each trial. Noise intensity level was calibrated using a Bruel&Kjaer (model 2250) sound-level meter to an average of 65 dB (dB) root-mean-square (r.m.s) level. Target tones were equated in intensity at a signal-to-noise-ratio of +2 dB relative to background intensity, based on the r.m.s level. The second tone was kept constant at 1024 Hz while the first varied pseudo-randomly over 7 (younger participants) or 5 (older participants) equally-spaced (on an octave scale) levels of frequency difference above or below the constant stimulus (pseudorandomized and balanced across all trials). These levels ranged from 0 Hz difference to 2Δ in younger and 2.5ΔHz in older participants (where Δ is the participants’ own 70% correct frequency difference limen). The reason we reduced the number of stimulus levels for the older adults was to keep the experimental duration to a minimum to avoid fatigue.

### 2.3 Experimental procedure

Auditory stimuli were controlled using MATLAB using the Psychophysics Toolbox Version 3 (Brainard, 1997) and presented using Sennheiser headphones. Prior to the main experiment, participants completed training trials to familiarize themselves with the task and their frequency difference (in noise) limens were obtained using three interleaved 2-down-1-up staircase procedures. In the actual experiment target tones were presented at one of six possible pseudorandom delays (2400 + n*33 ms, where n = 0 … 5) relative to background onset. Trials were separated by an inter-trial period uniformly distributed between 1.7 and 2.2s. Participants were instructed to respond as accurately as possible, and the background noise terminated once the response was provided, or after 4 s. Trials were presented in a block design of 120 trials per block, with each participant completing 360 trials in total.

### 2.4 EEG recording and pre-processing

EEG signals were recorded in a dark and electrically-attenuated room using an active 64-channel BioSemi system (BioSemi B.V., Netherlands). The electrooculogram (EOG) was derived from four electrodes placed at the outer canthi and below each eye. Electrode offsets were kept below 25 mV, and data were recorded at a 500 Hz sampling rate using a 208 Hz low-pass filter.

Pre-processing and data cleaning were carried out as described previously in Kayser et al. (2016). In brief, the data were filtered between 1 and 70 Hz and Independent Components Analysis was used to identify eye movement, blink artefacts (Debener et al., 2010) and muscle artefacts (Beirne and Patuzzi, 1999; Hipp and Siegel, 2013). Trials were rejected if the peak signal on any electrode exceeded ±100 μV. Further, trials were rejected if participants responded faster than 400 ms following the first target tone to ensure that participants had indeed listened to the entire available sensory evidence. Based on these criteria we rejected an average of 5% of trials. EEG signals were re-referenced to the common average for further analysis.

### 2.5 Analysis methods

#### 2.5.1 Evoked responses

We compute AEPs in response to the onset of the acoustic background based on trial-averaged data over a 3 × 3 grid of central channels (FC1, FCz, FC2, C1, Cz, C2, CP1, CPz, CP2). Individual participants’ P1, N1 and P2 component peak amplitude latencies and amplitudes were taken at the maximum negative or positive deflection within component-specific time windows. These time windows for finding subject-specific peaks (i.e. the maximum or minimum) were defined as follows, and differed between groups (Younger P1: 0–0.1 s; Younger N1: 0.08–0.2s; Younger P2: 0.15–0.35 s; Older P1: 0–0.1 s; Older N1: 0.05–0.2 s; Older P2 0.2–0.35s).

#### 2.5.2 Pre-target power spectra

Estimates of the frequency spectra of the ongoing EEG activity prior to the target stimuli were derived for each subject from a time window of −0.6 s to 0 s relative to target onset. To compute the spectral power we applied Welch's method to the concatenated the single trial data (using 600 ms Hanning windows; no overlap). PSD estimates were initially calculated for each channel and subsequently averaged. PSD estimates were normalized by removing individual participants' mean and PSD slopes were then fit in semi-log space using linear regression at frequencies between 1 Hz and 25 Hz whilst excluding alpha power between 7 and 14 Hz (Tran et al., 2016; Voytek et al., 2015).

#### 2.5.3 Single trial decoding of EEG signals

To link pre-stimulus activity with perception we used the same statistical modelling approach as in our previous study (Kayser et al., 2016). We computed pre-stimulus activity in task-relevant EEG components extracted using multivariate linear discriminant analysis (Boyle et al., 2017; Kayser et al., 2016; Parra et al., 2005; Philiastides, 2006; Ratcliff et al., 2009). We searched for discriminant components within the EEG data that best discriminated between the frequency conditions (i.e. 1st or 2nd tone higher in frequency). Each projection Y*(t)*, of the EEG data, *x(t)*, is defined by spatial weights, *w(t)*, and a constant, *c*, as follows:

$$\text{Y(t)}\;=\;\sum_{i}{\text{w}}_{\text{i}}{\text{x}}_{\text{i}}\text{(t)}\;+\;\text{c}$$

with *i* summing across channels. Classification was based on regularized linear discriminant analysis (Philiastides et al., 2014), which was applied to the EEG data in 80 ms sliding windows. We assessed classifier performance using the area under the receiver operator characteristic (ROC) curve (referred to herein as Az), based on 10-fold cross validation. The statistical significance of the performance was assessed by shuffling condition labels 1000 times, computing the group-average Az value for each randomization, and taking the maximal Az value along time to correct for multiple comparisons (Nichols and Holmes, 2003). We estimated the corresponding forward model for each component by computing the normalized correlation between the discriminating projection and the original EEG data (Parra et al., 2005).

To select scalp projections reflecting EEG activity that was temporally consistent across subjects, we selected three different components which corresponded to three continuous time windows using K-means clustering based on component topographies (see Kayser et al., 2016 for details). For each participant, we then extracted the weight (*w*) from the time point associated with the maximal Az value within each component for further analysis, which allowed us to incorporate between-subject variability in EEG timing in the analysis.

Since *Y(t)* is indicative of the extent of separability between frequency levels, we exploit this as a measure representing the amount of encoded sensory evidence about the task relevant tones (Grootswagers et al., 2017; Guggenmos et al., 2017). We computed each components’ time course by applying the respective weight to all trials and time points, resulting in a one-dimensional projection of single-trial task-related activity which we then analysed further.

#### 2.5.4 Pre-target time-frequency analysis

Time-frequency representations (TFRs) of the rhythmic brain activity prior to target were calculated using Morlet wavelets in FieldTrip (Oostenveld et al., 2011). Frequencies ranged from 2 Hz to 40 Hz in linear steps of 1 Hz below 16 Hz and 2 Hz above. To achieve greater frequency smoothing at the higher frequencies the width of individual wavelets scaled with frequency (min = 4 cycles, max = 9 cycles). TFRs were calculated between −0.6s and −0.1s relative to target onset in 50 ms bins. To avoid post-target contamination, we set the post-target period to zero for TFR analysis by applying a 40 ms Hanning window to the last 40 ms of the pre-stimulus period (Henry et al., 2014). For subsequent regression analyses, the power was z-scored within participants and frequency bands across time and trials (Kayser et al., 2016).

#### 2.5.5 Statistical analyses

Group-level psychometric curves were computed for the percentage of correct responses as a function of stimulus level (averaging over stimulus positions), and as a function of stimulus position (averaging over frequency difference). The median performance, averaging across stimulus levels and temporal positions, between age groups was compared using a Wilcoxon rank sum test, with effect size (r) calculated by dividing the Z-value by the square root of N, where N represents the number of observations (Field, 2013). To test whether performance differed as a function of stimulus position we used a non-parametric, one-way repeated-measures analysis of variance by ranks (Friedman Test).

AEP peak amplitudes/latencies and PSD slopes were compared between age groups using a non-parametric Wilcoxon rank-sum tests, with effect sizes (r) calculated following Field (2013).

To investigate the relationship between single-trial pre-stimulus activity (power/phase in particular frequency bands and time bins), sensory evidence, *Y(t)*, extracted from each component), and perceptual choice we used linear regression modelling (Fig. 4). Model 1 tested whether pre-stimulus power/phase influences choice using regularized logistic regression. Model 2 tested whether pre-stimulus power/phase influences sensory evidence *Y(t)* using linear regression. Model 3 tested for a direct influence of sensory evidence on choice. Finally, we tested for possible mediation effects, where pre-stimulus activity state influence choice through mediation of sensory evidence (i.e. an indirect influence of pre-stimulus state on choice; see Kayser et al., 2016) using an additional model: regression of choice on both *Y* and power/phase. Mediation effects were tested by comparing this with model 3. We calculated each model separately for power and phase, and for each pre-target time-frequency point. For regressions involving sensory evidence, we coded *Y(t)* as an unsigned variable and Z-scored this within each stimulus level, to reflect the amount of evidence about the respective stimulus. For phase, both sine- and cosine-transformed phase angles were submitted to the regression model. Mediation effects were defined by adjusting for dichotomous outcomes (MacKinnon et al., 2007).

Group-level statistical testing was performed using cluster-based permutation procedures (Maris and Oostenveld, 2007) and correcting for multiple comparisons across relevant dimensions, as described previously (Kayser et al., 2016). Specifically, we used 1000 randomizations, a 2.5th/97.5th percentile cut-off to define significant clusters, defining clusters by at least four significant neighbours, and using the cluster mass index. A two-sided test was performed on the clustered data and we corrected for multiple comparisons across regression models and components using the false discovery rate (FDR) at p < 0.05. We report effect sizes for clustering statistics as the cluster mass across all bins within a cluster (*T*_*sum*_).

The peak effect frequencies were compared across groups using a percentile bootstrap test (using 2000 samples). We randomly assigned participants to either group and compared the actual difference in group-level peak frequencies extracted from the respective statistical contrast for each regression factor to the distribution of differences in the randomized data. For this analysis effects were averaged over time for the duration of the respective clusters. Given that there were two significant clusters linking power to sensory evidence, we constrained the range of potential peak frequencies for each effect to distinct but overlapping ranges: for the alpha/beta cluster to 8–26 Hz, and for the low-frequency cluster to 2–13 Hz. We note that the results did not depend on the precise values of the respective cut-off frequencies.

**Fig. 2:**
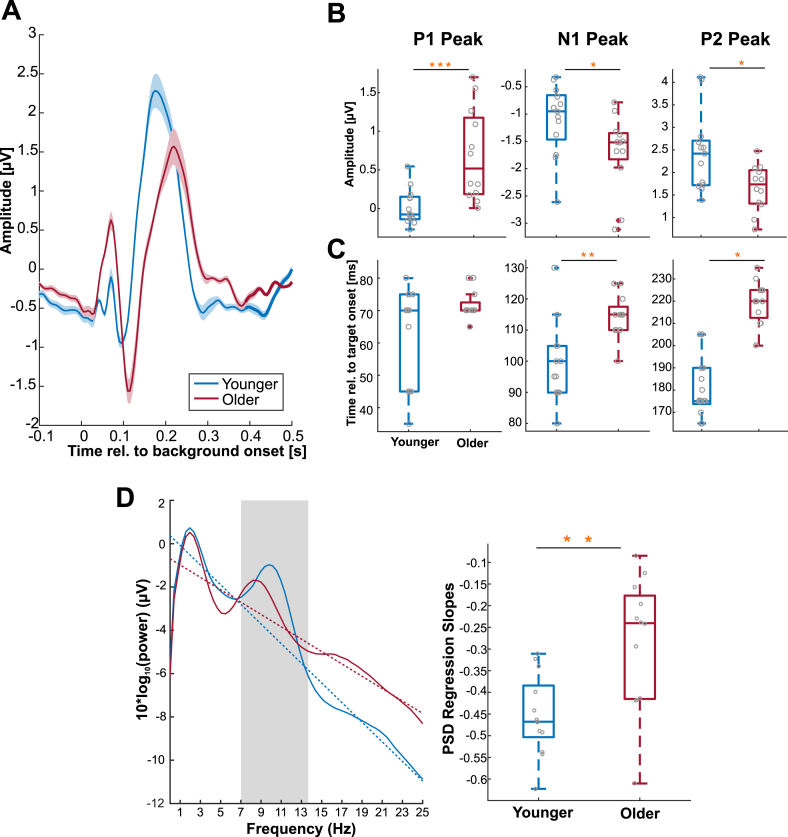
Auditory evoked responses to background onset and pre-stimulus power spectral density. (A) Grand-average AEPs with standard error (SEM) over central channels (FC1, FCz, FC2, C1, Cz, C2, CP1, CPz, CP2). Both younger and older subject display a clear P1-N1-P2 potential. (B) A comparison of AEP peak amplitudes between groups revealed an age-related enhancement of P1 and N1 peaks, and a reduction in the P2 peak. (C) Component peaks were also compared in terms of latencies, revealing an age-related delay in N1 and P2 peaks. (D left panel) Group-averaged PSD estimates (smooth curves) and fitted regression slopes (dashed lines) for frequencies up to 25 Hz, averaged over all channels. Slopes were computed whilst ignoring alpha power between 7 and 14 Hz (indicated by shaded area). (D right panel) PSD slopes were flatter for the older adults. Grey circles indicate individual subject data. Yellow asterisks indicate significance as follows: *p < 0.05, **p < 0.01, ***p < 0.001.

To link changes in AEP amplitudes and latencies to the peak frequencies of pre-stimulus effects we first computed leave-one-out estimates of the respective peak-frequencies of the pre-stimulus effects and of AEP amplitudes and latencies. We relied on a leave-out-one (Jacknife) approach as peak frequencies for pre-stimulus effects were more robust at the group-level than for individual subjects. We then used the six AEP characteristics (c.f. Fig. 2) as predictors for the peak frequency of the pre-stimulus effect across the full sample of younger and older participants in a linear regression model, for which we obtained the overall model performance and significance.

## 3 Results

### 3.1 Behavioural performance

As expected given the experimental design, the overall performance was comparable across group (averaged over stimulus level and target position younger median = 78.9% correct, older median = 74.4%, Z = 1.251, p = 0.211, r = 0.25; Fig. 1B). To avoid expectancy effects, target tone pairs were presented at six temporal positions relative to background onset. Friedman's tests revealed no effect of target position on performance in either group (younger adults: χ^2^(5) = 5.28, p = 0.382; older adults: χ^2^(5) = 8.3, p = 0.141; Fig. 1C). This suggests that any influence of pre-target activity on performance would occur without explicit entrainment of auditory cortical activity to the acoustic noise in either group (Henry and Obleser, 2012; Ng et al., 2012). Furthermore, this also rules out the possibility that the duration of the background sound prior to the target acted as a priming signal, the duration of which could have influenced performance.

### 3.2 Age-related changes in auditory evoked responses

To confirm previous reports of an age-related slowing of sensory-evoked activity we compared the latency and amplitude of evoked components (P1, N1 and P2; Fig. 2A). Peak amplitudes were significantly stronger for P1 and N1 in the older group, while P2 amplitudes were reduced (P1: younger median = −0.077 μV, older median = 0.517 μV, Z = -3.291, p = 9.991 × 10^−4^, r = −0.658; N1: younger median = −0.95 μV, older median = −1.519 μV, Z = 2.094, p = 0.0362, r = 0.419; P2: younger median = 2.418 μV, older median = 1.736μ, Z = 2.366, p = 0.018, r = 0.473; Fig. 2B). The latencies of N1 and P2 in the older adults were significantly delayed (N1: younger median = 0.1s, older median = 0.115s, Z = −2.99, p = 0.003, r = −0.598; P2: younger median = 0.175s, older median = 0.22s, Z = −4.124, p = 3.721 × 10^−5^, r = −0.825; Fig. 2C). There was no significant difference in P1 latency (younger median = 0.07s, older median = 0.07s, Z = −1.013, p = 0.311, r = −0.203).

### 3.3 Pre-target PSD flattens with age

Given previous reports of changes in the power spectra of ongoing brain activity with age (Klimesch, 1999; Tran et al., 2016; Voytek et al., 2015), we analysed the spectral slope of the EEG signal in the pre-target period (Fig. 2D). The PSD slopes of the older group were significantly flatter than those of the younger participants (younger median = −0.478 dB, older median −0.24 dB, Z = −2.91, p = 0.005, r = −0.582).

We also tested whether, across subjects, the observed changes in AEP latency and amplitude correlated with changes in spectral slope. Differences in PSD slope correlated significantly with differences in AEP latency for the P2 component (spearman rank-correlation: r = 0.42, p = 0.033, reduced slope corresponding to longer latency) but not the other AEP components (N1: r = 0.016, p = 0.93, P1: r = 0.165, p = 0.43). Differences in PSD slope also correlated with the amplitudes of the P1 (r = 0.43, p = 0.03) and N1 (r = −0.45, p = 0.025) peaks, with a flatter PSD spectrum correlating with stronger evoked responses. There was no correlation with the P2 amplitude (r = −0.25, p = 0.22).

### 3.4 Single trial decoding of EEG signals

**Fig. 3:**
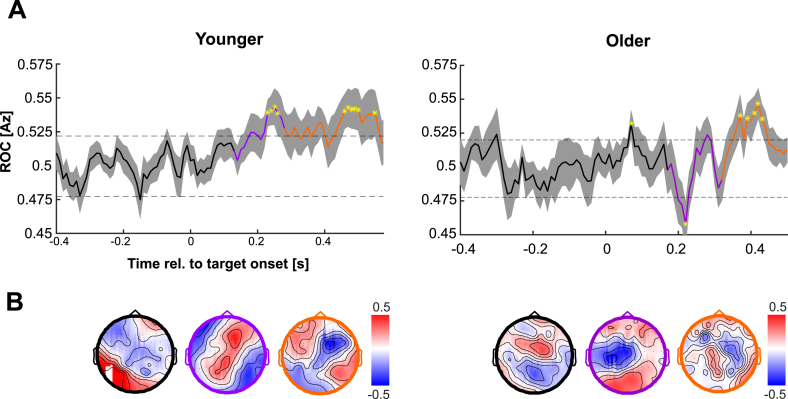
Task-relevant EEG components. (A) A linear classifier based on EEG data in 80 ms windows was used to discriminate between the two frequency conditions of interest. The smooth curve reflects group-averaged ROC values (Az) with SEM represented by shaded boundaries. Yellow asterisks highlight projections in which Az reached significance, and the dashed lines represents significance, based on randomisation tests (at p < 0.001). Coloured curve segments indicate the k-means clustering of scalp projections derived from the classifier topographies. Clustering revealed three distinct components, each systematically different temporally and topographically. The first cluster (black curve) spanned the epoch in which the stimulus was being presented; the second (purple) cluster comprises shorter-latency activity possibly originating from sensory-specific regions; and the third (orange) cluster reflects later-activated.

Using single-trial modelling we extracted EEG components that maximally differentiated between the stimulus conditions on which the participants task relied (1st or 2nd tone higher). For both groups, classification performance became significant around 0.2s following target onset (randomization test, p < 0.01, corrected for multiple comparisons along time, Fig. 3A).

**Fig. 4:**
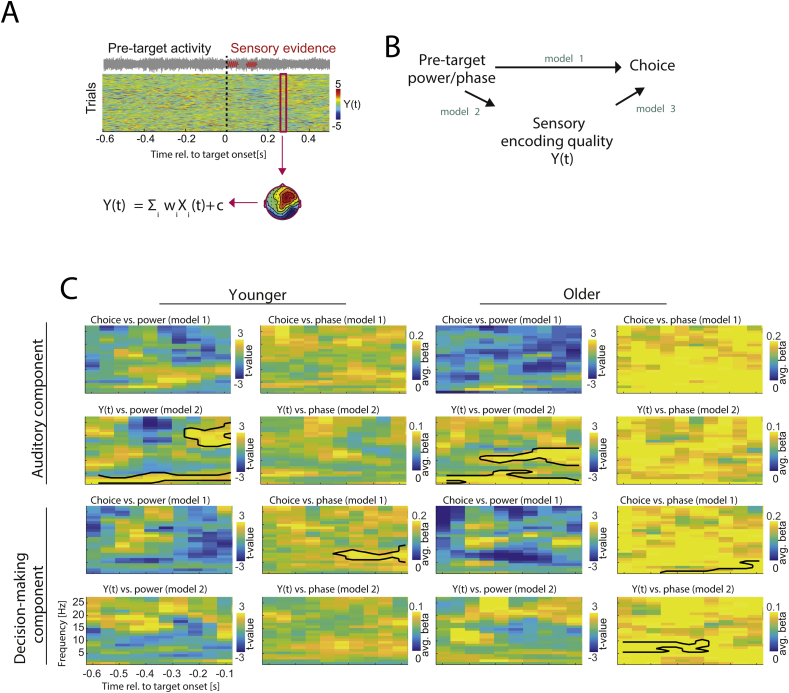
Linear modelling of pre-target activity on sensory evidence and choice within auditory and decision-making networks. (A upper panel) Single trial activity for one participant is shown. The red box highlights a classifier time window. (A lower panel) One-dimensional scalp projections carrying task relevant sensory evidence, *Y(t)* are derived from the single trial EEG data, *X(t)* and are defined by spatial weights, *w(t),* an a constant, *c*. (B) Models by which pre-stimulus activity could shape perceptual choice (c.f. Methods). (C) Group-level regression statistics for models 1 and 2, for both age groups and components. Significant time-frequency clusters are highlighted by black contours (at p < 0.05; FDR corrected across models and comparisons).

Using data-driven clustering based on individual subject's component topographies we extracted three temporally and topographically distinct component-clusters for each group (Kayser et al., 2016). For each of these clusters we derived the respective group-level topographies and classifier performance (Fig. 3B). Importantly, this analysis allowed us to incorporate inter-individual differences in the precise timing of relevant EEG activations, as within each of the three clusters, we selected for each subject the time point at which the respective discriminant component carried maximal information about the stimulus conditions.

The first EEG component spanned a time window encapsulating the majority of the stimulus presentation period (0s–0.15s) in both younger (0s–0.12s) and older (0s–0.16s) participants. Given the overlap with ongoing acoustic stimulus, this component was not considered further (see also Kayser et al., 2016). The second component (hereinafter termed the “auditory component”) spanned an early epoch (0.13s–0.28s in younger, and 0.17s–0.31s in older adults) and had a central topography in both groups. The third component (hereinafter termed the “decision-making component”) spanned a later epoch (0.28s–0.5s in younger, and 0.32s–0.5s in older adults). This late component likely reflects the transition between sensory encoding and perceptual decisions and was characterized by parieto-frontal topographies (Diaz et al., 2017; Giani et al., 2015; Marti et al., 2015). Both components significantly discriminated between frequency conditions in both age groups (ROC >0.5; randomization test, p < 0.01).

Noteworthy, while the overall topographic sequence of EEG components was the same across groups, the timing of each component was delayed by about 40 ms in the older group, matching the latency shift observed in the AEP P2 component.

### 3.5 Influence of pre-target activity within the auditory component

Having derived projections of single-trial task-related activity within meaningful EEG components we computed pre-target oscillatory activity for each of these (Fig. 4A). We then used statistical modelling to understand the tri-partite relation between pre-stimulus activity, the encoding of task-relevant information (as reflected by the EEG component) and behavioural choice (Fig. 4B). Specifically, we statistically tested the relations between power/phase (individually) and choice (model 1); power/phase and sensory evidence (model 2); and sensory evidence and choice (model 3).

For the auditory EEG component, we found no significant relation between pre-target power or phase on choice in either group (model 1; based on a significance level of p < 0.05, FDR corrected across models, Fig. 4C). However, there were significant relations between pre-target power and sensory evidence (model 2): in the younger group at low frequencies (2–6 Hz, −0.6s to −0.1s; T_sum_ = 66, p = 0.001) and the beta band (16–36 Hz, −0.3s to −0.1s, T_sum_ = 77, p = 0.002). The same effects were observed in the older group, albeit at slightly different frequencies: low frequency (2–7 Hz, −0.6s to −0.1s, T_sum_ = 128.6, p < 0.001) and alpha/beta band (10–16 Hz, −0.45s to −0.1s, T_sum_ = 9.6, p = 0.001). No relation between phase and sensory evidence was found in either group. The relation between sensory evidence and choice (model 3) was significant in the younger group (t(12) = 3.3, p = 0.006) and approached significance in older group (t(11) = 2.1, p = 0.054).

These results could be seen to suggest that pre-stimulus influences emerge systematically at lower frequencies in the older group. However, the existence of a significant cluster at a specific frequency does not demonstrate that this effect is significant only at that specific frequency. We hence used a bootstrap test to directly probe whether the group-level peak frequency for each cluster differed significantly between groups. This was not the case for either cluster (low frequency cluster: difference in peaks = 0 Hz, 95% bootstrap confidence interval (CI) [-9, +9] Hz, p = 0.199; alpha/beta cluster: difference in peaks = 7 Hz, CI [-13, +13] Hz, p = 0.173).

### 3.6 Influence of pre-target activity within the decision-making component

Repeating the same comparison for the late EEG component revealed a significant relation between pre-target phase and choice in the younger group around the alpha band (model 1; 7–14 Hz, −0.4s–0.1s, T_sum_ = 5, p = 0.003; Fig. 4C) and at low-frequencies in the older group (1–5 Hz, −0.4s to −0.1s, T_sum_ = 4.5, p = 0.003). Here we found some mild evidence that the respective peak frequencies may differ with age, as the difference was statistically significant (difference in peaks = 7 Hz, CI [-8,+9] Hz, p = 0.049).

Furthermore, there were no significant relations between power and choice in either group (model 1 for power) and there were no significant relations between power and sensory evidence (model 2). However, in the older group there was a significant relation between alpha phase and evidence (model 2; 8–12 Hz, −0.6s to −0.3s, T_sum_ = 3.1, p = 0.002), while no such effect was observed in the younger group. Additional mediation analysis revealed no significant mediation effects of phase on choice through evidence in either age group (at p < 0.05), and neither age group showed a significant relation between sensory evidence and choice (model 3; younger adults: t(12) = 1.1, p = 0.27; older adults: t(11) = 1.9, p = 0.0741), suggesting that the statistical relation between alpha phase and sensory evidence in the older group reflects a process not directly driving perceptual decisions.

Given that we found some evidence for pre-stimulus influences on choice to emerge at different frequencies in younger and older participants, we also asked whether this difference in peak frequency is related to the observed changes in amplitudes or latencies of the evoked potentials (c.f. Fig. 2). Specifically, we obtained leave-one-out estimates of the group-level peak frequencies for the pre-stimulus effects and AEP amplitudes/latencies in response to background onset. We then used these six AEP characteristics as predictors for the pre-stimulus peak frequencies across the sample of young and old participants. Together, the AEP characteristics provided significant predictive power (r^2^ = 0.81, F = 16.8, p < 10^−5^), suggesting that overall changes in the timing and amplitude of evoked responses with age are indeed related to the observed changes in relevant pre-stimulus frequencies.

## 4 Discussion

We have investigated the consistency of how pre-stimulus activity influences auditory frequency discrimination performance in young and older participants. In both groups the power of pre-stimulus activity influenced the encoding of sensory evidence reflected by early evoked components, while the phase influenced choice formation in later-activated EEG components. Importantly, for the early EEG components we did not find evidence for a systematic difference in the time scales of the perceptually relevant pre-stimulus activity between groups. In the later-activated EEG component we found a trend for perceptually relevant rhythmic activity to arise from slower frequencies in the aging brain. At the same time our data replicate previous findings of a significant age-related slowing of AEP latency, modulations of AEP amplitudes, and a flattening of the spectral profile of EEG activity.

### 4.1 Pre-stimulus influences on perception

In both groups we found that perceptual performance was influenced by rhythmic brain activity prior to the task-relevant stimulus. Our results hence confirm previous research showing that pre-stimulus brain activity influences perception in general (Florin et al., 2017; Henry et al., 2014; Henry and Obleser, 2012; Iemi et al., 2017; Kayser et al., 2016; Ng et al., 2012; Pinheiro et al., 2017; Samaha et al., 2017; Samaha and Postle, 2015; VanRullen, 2016).

In a previous study focusing on young subjects only we dissociated two mechanisms by which pre-stimulus activity influences auditory perception and mapped these onto distinct neural generators (Kayser et al., 2016). Specifically, we found that low frequency and alpha/beta power shaped the encoding of relevant sensory information in early-activated EEG components, which likely emerge from auditory cortical networks. In addition, the phase of alpha band activity emerging from later-activated fronto-parietal EEG components directly influenced the decision process. Here we replicated these results in a group of elderly participants characterized by no or mild hearing loss, in a paradigm where the overall task performance was equated between groups. Thereby the present data lend additional support to the hypothesis that multiple and distinct rhythmic processes control perceptual decisions and suggest that the relevant time scales of neural activity are largely conserved along the life span. Furthermore, they demonstrate that the relation of pre-stimulus brain activity and perception is not mandatorily affected by a general increase in neural response latencies with age.

### 4.2 Age-related changes in the timing of brain activity

In our data we found systematic age-related differences in the P1-N1-P2 evoked components of auditory evoked responses. Older adults’ P1 and N1 component amplitudes were significantly larger compared to younger adults, yet their P2 peaks were reduced. These findings are consistent with previous reports of age-related changes in AEP amplitude (Anderer et al., 1996; Czigler et al., 1992; Harkrider et al., 2005; Henry et al., 2017; Rufener et al., 2014; Tremblay et al., 2003), which may be attributed to age-related changes at the cellular level (Caspary et al., 2008; de Villers-Sidani et al., 2010; Hughes et al., 2010), neuronal synchrony (Anderson et al., 2012; Harris and Dubno, 2017), or changes in inhibitory control with age (Alain and Woods, 1999; Alain et al., 2014). Furthermore, we also found an age-related slowing of the N1 and P2 peak latencies, an effect consistently reported in ageing research (Henry et al., 2017; Tremblay et al., 2004).

We also found that the spectral profile of ongoing EEG activity was significantly flatter in the older participants. This is in line with previous reports which propose a mediating role of spectral flattening in cognitive decline (Tran et al., 2016; Voytek et al., 2015), possibly resulting from a decrease in neuronal synchrony (Podvalny et al., 2015; Pozzorini et al., 2013; Voytek and Knight, 2015; Waschke et al., 2017), increases in spontaneous activity (Hong and Rebec, 2012), or changes in the excitation inhibition balance (Caspary et al., 2008; Gao et al., 2017). Our participants passed a cognitive screening assessing a wide variety of cognitive abilities (reasoning, attention, working memory, abstraction, orientation, language), suggesting that the observed changes in spectral slope in the present data do not reflect cognitive decline itself but either compensatory mechanisms or basic changes in cellular physiology.

Previous EEG studies on stimulus-selective AEP components have suggested age-specific changes in the behavioural relevance of short- and long-latency components (Snyder and Alain, 2007, 2005). For example, so called object-related negativity potentials (ORN's) were found to consistently emerge at latencies of about 150 and 250 ms post-stimulus in younger and older listeners, but were absent at yet longer latencies in the elderly. Furthermore, perceptual performance was best predicted by ORN's at different latencies across age groups (Snyder and Alain, 2007, 2005). These findings are in contrast to the present study, where we consistently observed stimulus-selective discriminant components from short (around 150 ms) to long (up to 500 ms) latencies across age groups. This difference could result from methodological approaches: the previous studies used the same fixed EEG electrodes to compare ORN's between groups, while we performed electrode-wide classification analysis, which allows for different electrode configurations to yield stimulus-selective EEG components for each time point and subject. Our results thereby suggest that stimulus-selective EEG activations emerge at multiple latencies in both younger and older listeners, but may differ between groups in their precise timing or topographies.

### 4.3 Do pre-stimulus influences change with age?

Our main focus was on whether pre-stimulus influences on perception are comparable between young and older participants. While the statistical clusters of significant effects seemed to shift towards lower frequencies in the older group, direct statistical tests did not provide clear evidence for a systematic shift of pre-stimulus effects towards lower frequencies in the elderly. In particular, within the early-activated (“auditory”) EEG component there was no evidence for peak frequencies to differ between groups. Given a likely origin of this early EEG component in sensory-specific brain regions in the temporal lobe (Kayser et al., 2016), this suggests that the processes of early sensory encoding are conceptually conserved with age, despite a slowing of the respective evoked responses. Within the later-activated (“decision-making”) EEG component pre-stimulus effects on choice were more variable, and we observed a trend towards lower peak frequencies in the older group. This reduction in peak frequency was significantly related to changes in the timing and latency of evoked responses between groups. This later-activated EEG component likely captures high-level cognitive and decision making processes, as suggested by its longer latency relative to target onset and the fronto-parietal topography (Kayser et al., 2016). Our data hence suggest that pre-stimulus influences on auditory perception are largely conserved across the age span, but may become more variable with age for those processes reflecting higher-level cognitive processes (McGovern et al., 2017; Sander et al., 2012; Zanto and Gazzaley, 2014).

This conclusion is also supported by our finding that there was an influence of alpha phase on sensory evidence in the late EEG component that was significant only in the older group. This phase-effect did not directly influence subjects’ choice, and hence did not bear direct influence on behaviour. However, the stronger relation between alpha phase and sensory encoding may suggest that in the elderly subjects the encoding of the task-relevant sounds in fronto-parietal regions was affected by a reduced attentional commitment (Henry et al., 2017; Strauβ et al., 2014; Wöstmann et al., 2015, 2016). This reasoning is based on the notion that enhanced alpha power reflects reduced attention (Thut et al., 2012; Wöstmann et al., 2016) and the stronger selection of sensory information by modulating the excitability of sensory cortices (Iemi et al., 2017; Kayser et al., 2015; Strauβ et al., 2015). Increased alpha power is necessary to actually observe phase effects and hence the stronger phase-dependent gating of sound encoding in the elderly may reflect a reduced engagement of attention. In auditory perception, the enhancement of alpha activity is often inversely related to signal intelligibility and may reflect compensatory mechanisms during challenging listening conditions (Becker et al., 2013; Henry et al., 2017; McMahon et al., 2016; Obleser et al., 2012; Obleser and Weisz, 2012; Scharinger et al., 2014; Steinmetzger and Rosen, 2017; Wöstmann et al., 2015). Hence, differences in the relation of alpha activity and sensory encoding may reflect age-specific strategies of dealing with hearing in noise, and the underlying perceptual and cognitive strategies (McGovern et al., 2017).

One possibility, of course, is that the sample size in the present study was not sufficient to reveal systematic shifts in the relevant frequencies, or that such effects are smaller than the frequency resolution employed here. On the other hand, it could also be that the mechanisms and time scales by which pre-stimulus activity shapes sensory encoding remain indeed the same, despite an overall change in the relative amplitude of different frequency bands (Babiloni et al., 2006; Cummins and Finnigan, 2007; Rondina et al., 2016; Vlahou et al., 2014). Support for the latter conclusion comes from studies demonstrating a similar modulation of alpha band activity by acoustical structure and task demands in young and elderly participants (Erb and Obleser, 2013; Tune et al., 2018; Wöstmann et al., 2015), and from a study demonstrating a similar modulation of behavioural performance by stimulus-entrained delta-band activity in young and older participants (Henry et al., 2017). Furthermore, while many studies confirm age-related changes in the power of individual frequency bands with age, it remains unclear whether the peak frequencies of well-known brain rhythms change with age (Hong et al., 2015; Klimesch, 1999; McEvoy et al., 2001; Vlahou et al., 2014). In studies directly addressing such differences the effects are often at the edge of significance (Hong et al., 2015; McEvoy et al., 2001) or absent (Vlahou et al., 2014). As a result, further studies are required to more finely dissociate the various neural generators of pre-stimulus influences on perception in general, and their potential age-related changes in particular.

## 5 Conclusion

The present data demonstrate conceptually similar influences of rhythmic pre-stimulus activity on sensory encoding in young and older healthy listeners. This consistency in pre-stimulus effects arises largely despite systematic changes in the overall spectral profile of EEG activity and a general slowing of auditory evoked responses in the older participants, raising questions as to how these two processes are biophysically related. At the same time, we observed a trend towards a distinct influence of the timing of alpha and delta/theta band activity in later-activated EEG components with age, which calls for a more systematic assessment of the relation between rhythmic brain activity, sensory encoding and cognitive strategies in aging.
